# Relationship between proteinuria and changes in antepartum and postpartum choroidal thickness in patients with pre-eclampsia

**DOI:** 10.1038/s41598-024-63359-3

**Published:** 2024-06-03

**Authors:** You Hyun Lee, Do Yeon Kim, Jin Gon Bae, Yu Cheol Kim

**Affiliations:** 1 Department of Ophthalmology, Keimyung University School of Medicine, Daegu, Republic of Korea; 2 Department of Obstetrics, Keimyung University School of Medicine, Daegu, Korea

**Keywords:** Medical research, Signs and symptoms

## Abstract

Pre-eclampsia (PE) is a hypertensive disorder characterised by systemic vascular resistance and endothelial dysfunction. It is known to influence choroidal thickness (CT). No previous studies have explored the antepartum and postpartum changes in CT with respect to the protein-creatinine ratio (PCR), a measure of proteinuria that is a clinical hallmark of PE. This study evaluated the correlations between antepartum and postpartum CT and the PCR in patients with PE. In this retrospective study, sixty-six eyes (66 patients) were analysed. The patients were divided into two groups according to the median PCR value (2.36 mg/mg): low PCR group (< 2.36 mg/mg) and high PCR group (≥ 2.36 mg/mg). Ophthalmologic clinical data were collected and assessed. We observed higher antepartum CT and higher mean arterial pressure in high PCR group than in low PCR group. Moreover, postpartum CT decreased significantly in high PCR group. In the multivariate analysis, CT changes were correlated with antepartum CT and antepartum PCR after logarithm transformation. In conclusion, a greater decrease in CT was observed in high PCR group than in low PCR group. Further, the antepartum PCR showed a correlation with the extent of CT reduction.

## Introduction

Pre-eclampsia (PE) is a complex hypertensive disorder known for its significant contribution to maternal and neonatal morbidity and mortality^1–3^. The underlying pathophysiology of this disease remains unclear; however, it is associated with a compromised maternal vascular response to placentation, leading to increased systemic vascular resistance and endothelial dysfunction^4^.

The choroid is one of the most highly vascularised tissues; it has the highest blood flow-to-tissue volume ratio. Its sensitivity to systemic changes makes it vulnerable to PE, in which fluctuations in circulating anti-angiogenic factors induce endothelial dysfunction, consequently affecting choroidal thickness (CT)^5–7^.

Previous research has established that patients with PE have higher CT than their healthy pregnant counterparts^8,9^. Further examinations by Kim et al.^8^ and Duru et al.^10^ compared antepartum and postpartum CT in patients with PE, revealing a notable reduction in CT postpartum.

Proteinuria, a clinical hallmark of PE, is associated with maternal and foetal morbidities^11^. The spot (random) urine protein-creatinine ratio (PCR, with a normal range of < 0.3 mg/mg) is typically used to assess quantitative proteinuria^12^. Our previous work indicated a correlation between CT and the PCR^13^; however, to the best of our knowledge, no studies have explored the relationship of antepartum and postpartum CT with the PCR. Therefore, this study aimed to evaluate the correlation between antepartum and postpartum CT and the PCR in patients with PE.

## Results

### Participants’ demographics and clinical characteristics

A total of 66 eyes (66 patients) were included in this study. The demographic data and clinical characteristics of low and high PCR groups are presented in Table 1. The mean antepartum PCR was 1.03 ± 0.69 in group 1 and 4.48 ± 1.96 in high PCR group. The postpartum PCR was significantly higher in high PCR group (3.22 ± 2.77 vs. 0.92 ± 0.42, p < 0.001), with a more significant decrease observed in high PCR group (− 1.57 ± 2.13 vs. − 0.23 ± 0.57, p < 0.001). A significantly higher antepartum mean arterial pressure (MAP) was observed in high PCR group than in low PCR group (124.71 ± 14.66 vs. 117.40 ± 11.72, p = 0.029). A greater decrease in postpartum MAP was also noted in high PCR group (− 6.32 ± 16.66 vs. − 3.63 ± 9.94, p = 0.042). Additionally, high PCR group had a lower mean body mass index (28.26 ± 4.84 vs. 29.77 ± 4.68), higher mean gestational age at delivery (33.50 ± 3.91 vs. 32.53 ± 2.63), higher percentage of use of blood pressure-lowering agents (82.4% vs. 65.6%), higher foetal growth retardation rate (382% vs. 25.0%), and lower mean baby birth weight (1833.65 ± 709.45 g vs. 2133.753 ± 682.24 g) than low PCR group, although these results were not statistically significant (p > 0.05). The clinical characteristics, such as best-corrected visual acuity, intraocular pressure, spherical equivalent, and choroidal vascularity index (CVI), also showed insignificant differences between the two groups (p > 0.05).

**Table 1:** Demographics and clinical characteristics.

	Low PCR group (n = 34)	High PCR group (n = 32)	*p*-value*
Age (years)	33.50 ± 3.90	34.68 ± 4.60	0.268
BCVA (LogMAR)	0.03 ± 0.09	0.05 ± 0.13	0.526
IOP (mmHg)	14.44 ± 3.43	14.68 ± 3.13	0.768
SE (D)	− 1.58 ± 1.50	− 1.93 ± 2.05	0.436
Choroidal vascular index	0.69 ± 0.31	0.71 ± 0.05	0.286
Body mass index (kg/m^2^)	29.77 ± 4.68	28.26 ± 4.84	0.202
GA at delivery (weeks)	32.53 ± 2.63	33.50 ± 3.91	0.240
Antepartum MAP (mmHg)	117.40 ± 11.72	124.71 ± 14.66	0.029*
Postpartum MAP (mmHg)	115.31 ± 10.92	119.24 ± 16.12	0.155
MAP changes (mmHg)	− 3.63 ± 9.94	− 6.32 ± 16.66	0.042*
Antepartum PCR	1.03 ± 0.69	4.48 ± 1.96	0.000*
Postpartum PCR	0.92 ± 0.42	3.22 ± 2.77	0.000*
Protein-creatinine ratio changes	− 0.23 ± 0.57	− 1.57 ± 2.13	0.000*
BP-lowering agent use (%)	65.6	82.4	0.120
Foetal growth retardation (%)	25.0	38.2	0.249
Baby birth weight (g)	2133.753 ± 682.24	1833.65 ± 709.45	0.089

*P-value was calculated using the independent Student’s *t*-test.

*BCVA* best-corrected visual acuity, *logMAR* logarithm of the minimum angle of resolution, *IOP* intraocular pressure, *SE* spherical equivalent, *GA* gestational age, *MAP* mean arterial pressure, *PCR* protein-creatinine ratio, *BP* blood pressure.

### Comparison of CT and serum angiogenic factors between the two groups

#### CT changes

The antepartum CT was significantly higher in high PCR group compared to low PCR group, as shown in Table 2 (262.44 ± 58.82 vs. 224.44 ± 49.12, p = 0.006). However, no significant difference was observed in postpartum CT between the two groups (223.34 ± 53.36 vs. 248.41 ± 73.11, p = 0.101). Notably, high PCR group experienced a significant reduction in CT postpartum (p = 0.015), while changes in low PCR group were minimal (p = 0.748). The decrease in postpartum CT was substantially larger in high PCR group compared to low PCR group (− 26.44 ± 32.36 vs. − 0.34 ± 19.01, p < 0.001).

**Table 2:** Comparison of the choroidal thickness.

	Low PCR group (n = 34)	High PCR group (n = 32)	*p*-value*
Antepartum CT (µm)	224.44 ± 49.12	262.44 ± 58.82	0.006*
Postpartum CT (µm)	223.34 ± 53.36	248.41 ± 73.11	0.101
*p*-value of the within-group comparison of the antepartum and postpartum CT	0.748	0.015**	
CT change (µm)	− 0.34 ± 19.01	− 26.44 ± 32.36	0.000*

*P-value was calculated using the independent Student’s *t*-test.

**P-value was calculated using the paired sample *t*-test.

*CT* choroidal thickness.

#### Serum angiogenic factors

As detailed in Table 3, high PCR group showed significantly higher levels of soluble fms-like tyrosine kinase-1 (sFlt-1) compared to group 1 (21,635.83 ± 13,248.98 vs. 6654.74 ± 5300.62, p < 0.001) and significantly lower levels of placental growth factor (PlGF) (48.97 ± 29.86 vs. 177.09 ± 151.23, p = 0.003). Consequently, the sFlt-1/PlGF ratio was markedly higher in high PCR group (610.58 ± 428.30 vs. 77.11 ± 76.27, p < 0.001), indicating a significant disparity in angiogenic balance between the groups.

**Table 3:** Comparison of the serum angiogenic factors.

	Low PCR group (n = 17)	High PCR group (n = 24)	*p*-value*
sFlt-1	6654.74 ± 5300.62	21,635.83 ± 13,248.98	0.000*
PlGF	177.09 ± 151.23	48.97 ± 29.86	0.003*
sFlt-1/PlGF ratio	77.11 ± 76.27	610.58 ± 428.30	0.000*

*P-value was calculated using the independent Student’s *t*-test.

*sFlt-1* soluble fms-like tyrosine kinase-1, *PlGF* placental growth factor.

### Factors associated with the antepartum PCR and CT changes

#### Antepartum PCR and associated factors

**Figure 1:**
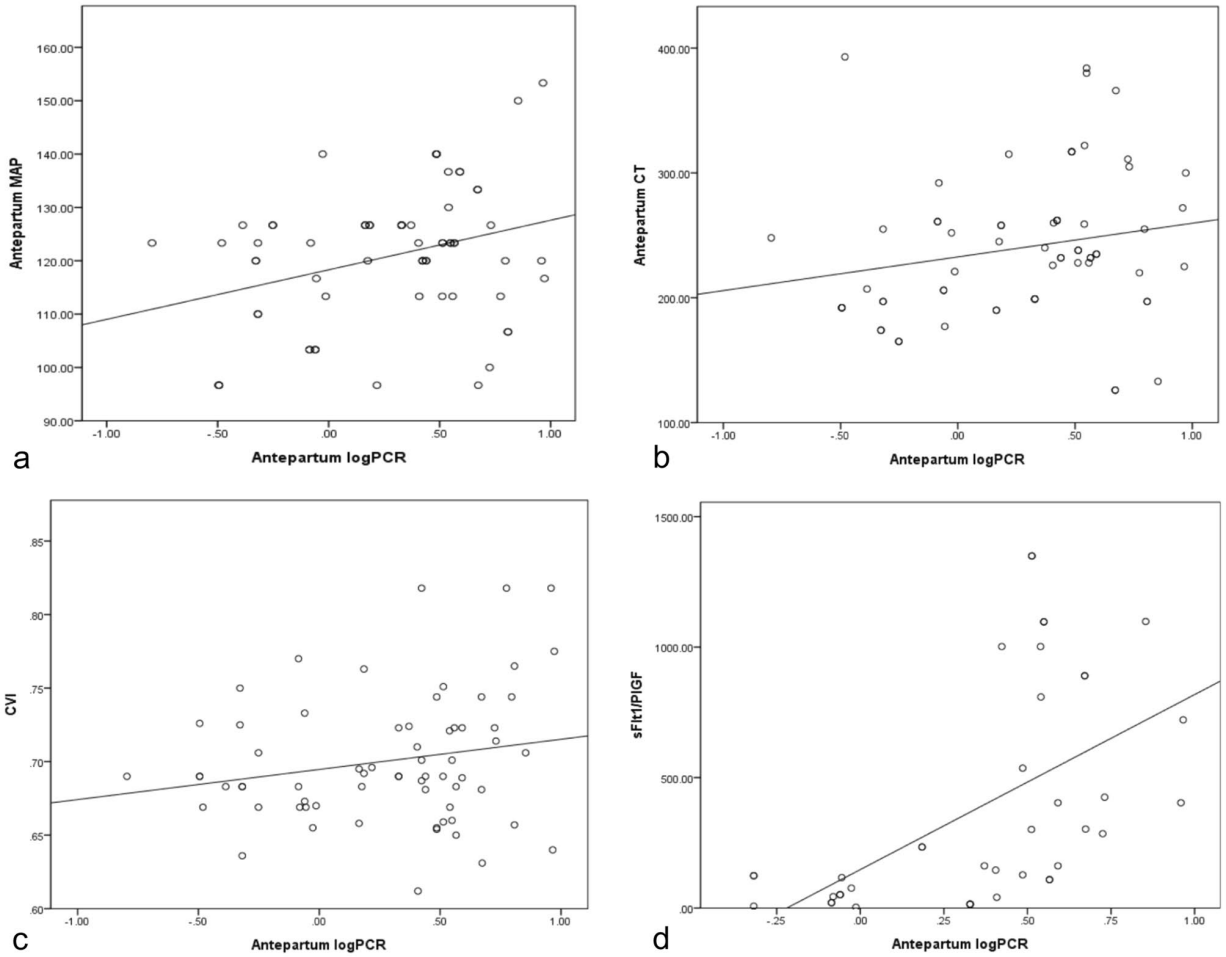
Correlations between the antepartum MAP, antepartum logPCR, sFlt-1/PlGF ratio, and antepartum CT. (**a**–**d**) Antepartum logPCR correlations with antepartum MAP, antepartum CT, CVI, and sFlt1/PIGF with a linear fit line. The correlation values are (**a**) r = 0.316, p = 0.010, (**b**) r = 0.293, p = 0.017, (**c**) r = 0.204, p = 0.100, and (**d**) r = 0.551, p < 0.001. *logPCR* protein-creatinine ratio in urine after logarithm transformation, *MAP* mean arterial pressure, *CT* choroidal thickness, *CVI* choroidal vascularity index, *sFlt-1* soluble fms-like tyrosine kinase-1, *PlGF* placental growth factor.

A logarithmic transformation was applied to PCR values to perform the correlation analysis (logPCR), illustrated in Fig. 1. This analysis revealed significant correlations: antepartum CT was correlated with antepartum logPCR (r = 0.293, p = 0.017); there was a correlation between the sFlt1/PIGF ratio and antepartum logPCR (r = 0.551, p < 0.001); and a correlation was also observed with antepartum mean arterial pressure (MAP) (r = 0.316, p = 0.010). The choroidal vascularity index (CVI), however, did not show a significant correlation (r = 0.204, p = 0.100).

#### Analysis of CT changes

The relationships between CT changes and other variables are detailed in Table 4. Univariable analysis showed significant correlations with antepartum logPCR (r = − 0.414, p = 0.001), logPCR changes (r = 0.212, p = 0.031), the sFlt1/PIGF ratio (r = − 0.333, p = 0.033), and antepartum CT (r = − 0.454, p < 0.001). Multivariable analysis further supported significant correlations with antepartum logPCR (r = − 0.591, p = 0.006) and antepartum CT (r = − 0.396, p = 0.019), indicating that these factors play a significant role in CT changes during the studied period.

**Table 4:** Factors associated with choroidal thickness changes.

	Univariable linear regression analysis		Multivariable linear regression analysis	
	Coefficient	P-value	Coefficient	P-value
Age	0.826	0.604	0.527	0.477
BMI	− 2.425	0.126	− 1.334	0.249
Antepartum logPCR	− 0.414	0.001*	− 0.591	0.006**
logPCR changes	0.212	0.031*	0.144	0.187
sFlt-1/PlGF ratio	− 0.333	0.033*	− 0.201	0.194
Antepartum MAP	− 0.083	0.509		
MAP changes	0.032	0.411		
Antepartum CT	− 0.454	0.000*	− 0.396	0.019**
Choroidal vascular index	0.028	0.825		

*Statistically significant based on the univariable linear regression analysis.

**Statistically significant based on the multivariable stepwise regression analysis using the backward elimination method (R^2^ = 0.531).

*logPCR* protein-creatinine ratio in urine after logarithm transformation, *sFlt-1* soluble fms-like tyrosine kinase-1, *PlGF* placental growth factor, *MAP* mean arterial pressure, *CT* choroidal thickness.

## Discussion

In the current study, we retrospectively analysed the correlation between proteinuria and changes in CT in patients with PE. We found that CT changes correlated with antepartum logPCR and antepartum CT.

In patients with PE, the vascular endothelial function is usually reduced, which increases the permeability of the glomerular basement membrane to serum proteins and albumins^14^. Proteinuria, a consequence of enhanced glomerular permeability, is a significant diagnostic criterion for PE. Dong et al.^15^ reported that proteinuria is closely related to the severity of PE. The PCR is widely used to assess the amount of proteinuria and the function of the glomerular cells^12,16^. In the current study, we observed a significantly high antepartum MAP and, although not statistically significant (p = 0.089), a lower baby body weight in subjects with high PCR than in subjects with low PCR. We also found that the antepartum logPCR correlated with the antepartum MAP. These findings suggest that the antepartum PCR may indicate disease severity in patients with PE.

The choroid, which consists of fenestrated capillaries and is controlled by autonomic nerves, is sensitive to changes in PE. Previous studies have reported higher CT in pregnant women with PE than in those without it^8,17,18^. The mechanism underlying increased CT in PE is considered to be vascular endothelial cell dysfunction^18^. However, Levine et al.^19,20^ reported an upregulation of anti-angiogenic proteins, such as sFlt1 and soluble endoglin, in women predisposed to developing PE. It has been postulated that these proteins interact with PIGF and vascular endothelial growth factor, culminating in an anti-angiogenic state best represented by the sFlt1/PlGF ratio. This anti-angiogenic environment contributes to endothelial cell apoptosis. This apoptosis has been found to be positively correlated with the sFlt1/PlGF ratio, which is considered indicative of endothelial damage^21^. In support of these findings, our previous study established a relationship between antepartum logPCR and the sFlt1/PlGF ratio with CT in women with PE^13,22^. This relationship is further corroborated by the results of the present study.

The increased CT in the PE group returned to normal after delivery. Duru et al.^10^ and Kim et al.^8^ found significant decreases in CT after delivery in patients with PE. Consistent with these findings, we also observed a decrease in postpartum CT in both groups; however, a statistically significant decrease was observed only in subjects with high PCR. Furthermore, a significantly greater decrease in postpartum CT was observed in subjects with high PCR than in subjects with low PCR. Petrozella et al.^21^ reported that endothelial damage in patients with PE persists for up to a week after delivery. The mean interval between the antepartum and postpartum periods in our study was 2.3 days. Thus, the rapid decrease in postpartum CT cannot be solely attributed to the restoration of endothelial cell function. It has been suggested that nephrotic proteinuria in patients with PE initiates intricate neuroendocrine mechanisms, leading to sympathetic overactivity. This overactivity can alter the systemic blood flow, resulting in CT changes^23^. Our study also revealed that the antepartum logPCR and antepartum CT were correlated with CT changes in the multivariate analysis. Based on these findings, we propose that early postpartum CT alterations are more strongly affected by the sympathetic outflow caused by proteinuria.

In light of our findings, it is evident that PCR values serve as significant predictors of CT changes in patients with PE, suggesting that those with higher PCR levels experience more pronounced postpartum CT reductions. This association underscores the importance of stratified patient management and monitoring, especially for those exhibiting higher PCR levels, as these changes suggest more than just endothelial repair. Rapid CT changes post-delivery likely involve complex neuroendocrine responses that merit further investigation. Understanding these mechanisms could lead to more targeted and effective management strategies, potentially improving both maternal and fetal outcomes. Furthermore, the pronounced CT changes in patients with elevated PCR values highlight the need for ongoing research into the underlying pathophysiological mechanisms, which could pave the way for novel therapeutic approaches in managing PE.

This study had certain limitations. The serum angiogenic factors were not fully assessed in all participants, and the sample size may have been insufficient to confirm the observed correlations in these variables. Although we collected the data of patients with PE in a certain trimester, the gestational age (GA) varied within that trimester and was not strictly controlled. Given that the choroid is sensitive to GA^7,24^, the CT may have differed significantly in each patient. Moreover, despite the high resolution of the swept-source optical coherence tomography (OCT), acquiring high-quality images of a thickened choroid was challenging, which led to underestimated values or null results. Future prospective studies should incorporate larger participant cohorts and more comprehensive assessment of clinical characteristics, including the presence of chronic hypertension and the timing of preeclampsia onset (early or late), to provide insights of greater clinical relevance and robustness.

In conclusion, a greater decrease in CT was observed in subjects with high PCR than in subjects with low PCR. Moreover, the antepartum PCR showed a correlation with the extent of CT reduction.

## Methods

### Study design and participants

This retrospective, comparative case series was conducted at a single hospital. The study adhered to the tenets of the Declaration of Helsinki, and the study design was approved by the Institutional Review Board of Keimyung University Dongsan Hospital (IRB no. 2023-06-002). The need for informed consent was waived due to the retrospective nature of the study by the Institutional Review Board of Keimyung University Dongsan Hospital (IRB no. 2023-06-002).

We retrospectively reviewed the electronic medical records of patients with PE who were referred to the ophthalmology department during hospitalisation for delivery between October 2018 and December 2022. The exclusion criteria were as follows: (1) smoking, (2) history of cardiac disease, (3) history of kidney disease that could affect renal function, (4) refractive error exceeding − 6.0 D, (5) history of pars plana vitrectomy, (6) presence of other ophthalmic diseases, such as diabetic retinopathy, rhegmatogenous retinal detachment, age-related macular degeneration, and uveitis, and (7) history of retinal photocoagulation.

Data regarding the GA, body mass index (kg/m^2^), and usage of blood pressure-lowering agents were recorded. Additionally, data on antepartum and postpartum MAP, PCR, and OCT images were collected based on ophthalmologic examinations conducted within two weeks before and after the delivery date. The antepartum and postpartum MAPs were assessed during the ophthalmologic examination. Antepartum serum levels of sFlt-1 and PlGF were analysed using standard serum tubes. Samples were centrifuged and subsequently stored at − 20 °C until analysis. Concentrations of sFlt-1 and PlGF were measured using electrochemiluminescence immunoassay on a Roche Diagnostics Cobas e602 system (Roche Diagnostics, Mannheim, Germany). The sFlt-1/PlGF ratio was then calculated based on these measurements.

The diagnosis of PE adhered to the standards established by the National High Blood Pressure Education Programme Working Group of the National Institutes of Health. The condition was characterised by an elevation in blood pressure to at least 140/90 mmHg after the 20th week of pregnancy in a woman with a prior normotensive state, accompanied by the onset of proteinuria (≥ 300 mg/24 h).

Patients were allocated into two groups based on the median PCR values: those with a PCR less than 2.36 mg/mg were categorized as the low PCR group, and those with a PCR of 2.36 mg/mg or greater were categorized as the high PCR group.

### Ophthalmologic clinical data collection

All patients underwent comprehensive ophthalmic assessment, including best-corrected visual acuity (logarithm of the minimum angle of resolution) measurement, intraocular pressure measurement using Goldmann applanation tonometry (AT 900^®^, Haag-Streit, Koniz, Germany), spherical equivalent assessment, and slit-lamp examination of the anterior and posterior segments.

OCT images were acquired using swept-source OCT (DRI OCT Triton^®^, Topcon, Tokyo, Japan) by a skilled examiner in the afternoon (12:00–5:00 pm) to avoid diurnal variations in the choroid^25,26^. The OCT images were acquired on the day of admission and during the postpartum period (median postpartum day 2, range: 1–4 days). The best OCT image of each eye with a clear posterior margin of the choroid was chosen for analysis. The average choroidal thickness in the Early Treatment Diabetic Retinopathy Study grid was automatically measured using a thickness map of the macula from a raster scan. The CVI was calculated to explore the vascular regions in the choroid from transfoveal horizontal OCT scans according to the method suggested by Agrawal et al.^27^.

### Statistical analyses

Data are presented as means ± standard deviations or number (percentage) of eyes. A total of 66 study participants (44 eyes) were included in this study. To avoid potential issues of skewed data, a logarithmic transformation was applied to the PCR values, enabling the exploration of a linear relationship with the ocular measurement values.

Statistical analyses were performed using Statistical Package for the Social Sciences (version 12.0; IBM Corp., Armonk, NY, USA). The categorical values of the demographics and clinical characteristics of the two groups were compared using Pearson’s Chi-square test. Continuous values of the two groups were compared using independent Student’s *t*-tests. Antepartum and postpartum CT were compared within groups using the paired sample *t*-test. A *p*-value < 0.05 was considered statistically significant. In our assessment of factors associated with CT changes, we initially conducted univariable linear regression analyses. Factors that demonstrated a *p*-value < 0.05, along with potential confounding variables such as age and BMI, were then included in a multivariable regression analysis. We employed the backward elimination method to effectively control for potential confounders.

## Data Availability

The datasets generated and/or analysed during the present study are available from the corresponding author on reasonable request.
